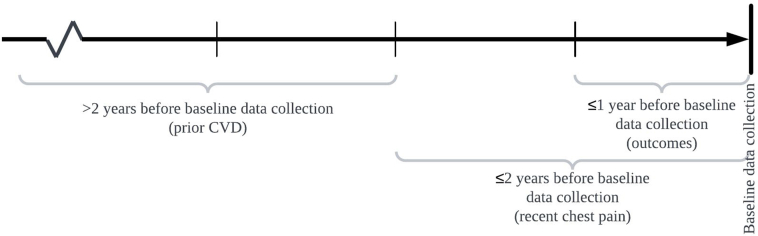# Chest pain in a multi-ethnic population: A community-based study on sex differences in chest pain prevalence and care contacts^☆^

**DOI:** 10.1016/j.ijcrp.2024.200361

**Published:** 2024-12-17

**Authors:** Bryn Hummel, Ralf E. Harskamp, Annick Vester, Henrike Galenkamp, Paula M.C. Mommersteeg, Irene G.M. van Valkengoed, the IMPRESS consortium

**Affiliations:** a Department of Public and Occupational Health, Amsterdam UMC, Location AMC, Amsterdam, the Netherlands; b Department of General Practice, Amsterdam UMC, Location AMC, Amsterdam, the Netherlands; c Center of Research on Psychological Disorders and Somatic Diseases (CoRPS), Department of Medical and Clinical Psychology, Tilburg University, Tilburg, the Netherlands

**Keywords:** Chest pain, Sex differences, Ethnicity, Healthcare seeking behaviour

## Abstract

**Objective:**

While chest pain is a common symptom, its prevalence among women and men across ethnic groups is unknown. Moreover, how chest pain is associated with general practitioner (GP) and cardiologist visits in women and men across ethnic groups, remains to be determined.

**Design:**

We used baseline data on 12423 women and 9071 men from the multi-ethnic HELIUS cohort (Amsterdam, the Netherlands; 2011–2015). Using logistic regressions, we studied sex differences in chest pain prevalence across ethnic groups. Next, in those who reported chest pain in the past two years (henceforth; recent chest pain), we studied sex differences in GP, cardiologist, and any specialists visits, in total and by ethnicity. Analyses were adjusted for age, ethnicity (in the total population), socioeconomic factors, associated symptoms, clinical parameters, and lifestyle factors.

**Results:**

Across most ethnic groups, women were more likely than men to report lifetime (33 % vs 29 %, p < .001) and recent chest pain (4.5 % vs 2.7 %, p = .001). In those with recent chest pain, women were more likely to have visited a GP, yet less likely to have visited a cardiologist, but not any specialist, compared to men. These differences were also observed in several sensitivity analyses, including in those with symptoms suggestive of typical Angina Pectoris.

**Conclusion:**

Chest pain is more commonly reported in women than men across most ethnic groups. While men were less likely to have visited a GP than women, women were less likely to have visited a cardiologist. Combined, this suggests delays in care may occur at different points in the care trajectory for women and men.

## 1 Introduction

With an estimated 20–40 % of the general population experiencing chest pain in their lifetime [1], chest pain is a common symptom. Chest pain is often a transient phenomenon, but -when persistent-may be associated with poor health and quality of life. The etiology varies from common mild conditions, e.g. chest wall pain, to potentially life-threatening conditions, such as an acute coronary syndrome or pulmonary embolism [[2], [3], [4]], which are less common in general practice. Due to its various nature and frequent occurrence, chest pain is a common reason to seek medical attention: an estimated 0.7–2.7 % of general practitioner (GP) consultations concern chest pain [[5], [6], [7], [8], [9]].

Studies suggests the prevalence of chest pain in the general population is higher in women than men [10]. Moreover, more consultations for chest pain are recorded for women than men in general practice [8], which -besides sex differences in prevalence of chest pain-may reflect sex differences in healthcare seeking behaviour (HCSB). Furthermore, evidence also suggests sex differences in referrals to specialists, as men with chest pain may be more likely to receive referrals to a cardiologist than women experiencing similar symptoms [11]. However, evidence on sex differences in the prevalence of chest pain and subsequent care contacts is scarce, and mostly limited to majority white populations, despite evidence suggesting ethnic differences in the prevalence of chest pain [12], cardiovascular risk profiles [13] and HCSB [[14], [15], [16], [17]].

Understanding sex- and ethnic variation in the prevalence of chest pain and HCSB is important, as not (timely) seeking GP care is associated with delayed diagnostics and referrals to specialist care, and ultimately poorer prognosis, in particular for those at risk of cardiovascular disease (CVD). Hence, in this study, we first analyzed sex differences in the prevalence of chest pain in a multi-ethnic population, aged 18–70 years, in Amsterdam, the Netherlands. Second, in those with chest pain, we studied sex differences in care contacts, specifically GP, cardiologist, and any specialist visits.

## 2 Materials and methods

### 2.1 Study design and data collection

We conducted a cross-sectional analysis using baseline data, collected between 2011 and 2015, from the population-based HEalty LIfe in an Urban Setting (HELIUS) study. This is a cohort of individuals of Dutch, Surinamese, Ghanaian, Turkish, and Moroccan origin living in Amsterdam, the Netherlands. Baseline data were obtained through a questionnaire, physical examinations, and analysis of biological samples. Additionally, participants were asked to bring their prescribed medication to their appointment, to be classified according to Anatomical Therapeutic Chemical (ATC) codes. A complete description of HELIUS is available elsewhere [18]. All participants provided written informed consent and the HELIUS study was approved by the Institutional Review Board of the Academic Medical Centre. The definitions used are described in brief below and more extensively in Supplemental Table 1.

### 2.2 Chest pain

Chest pain was measured using the Rose Angina Questionnaire, and we distinguish between lifetime chest pain (any self-reported chest pain), ‘recent chest pain’ (chest pain within the two years before baseline), and typical Angina Pectoris (AP) [19].

### 2.3 Outcomes

Our primary outcomes were self-reported recent visits to a GP, a cardiologist, and any specialist [4]. Any specialist included consultations with cardiologists, pulmonologists, internists and/or gastroenterologists, and mental health specialists during regular visits or emergency department visits. As a secondary outcome, we included supplementary diagnostics for identifying cardiovascular risk that may be assessed when AP is suspected [19].

### 2.4 Covariates

#### 2.4.1 Sociodemographic variables

Age (in years) and sex (women and men) were derived from the municipality registry.

Ethnicity was defined according to participants' and their parents' country of birth [18]. Surinamese participants were further classified into ‘South Asian’, ‘African’, ‘Javanese’ or ‘other’, based on self-report.

#### 2.4.2 Socioeconomic factors

Socioeconomic status (SES), commonly defined based on educational level and employment status, may be associated with access to healthcare [20]. Both measures were dichotomised due to the low numbers of participants in the ‘recent chest pain’ subgroup.

#### 2.4.3 Associated symptoms

As the presence of associated symptoms in those with chest pain may relate to HCSB and referrals, we adjusted for dyspnoea and fatigue.

#### 2.4.4 Clinical parameters

Because comorbidities may associate with HCSB and referrals in those with chest pain, we included awareness of clinical parameters (hypertension, diabetes, hypercholesterolemia), depressive symptoms [21], and prior diagnoses [22] (Supplemental Fig. 1).

#### 2.4.5 Lifestyle factors

As lifestyle factors may also be associated with HCSB [14], we adjusted for obesity and smoking status.

### 2.5 Study sample

**Fig. 1:**
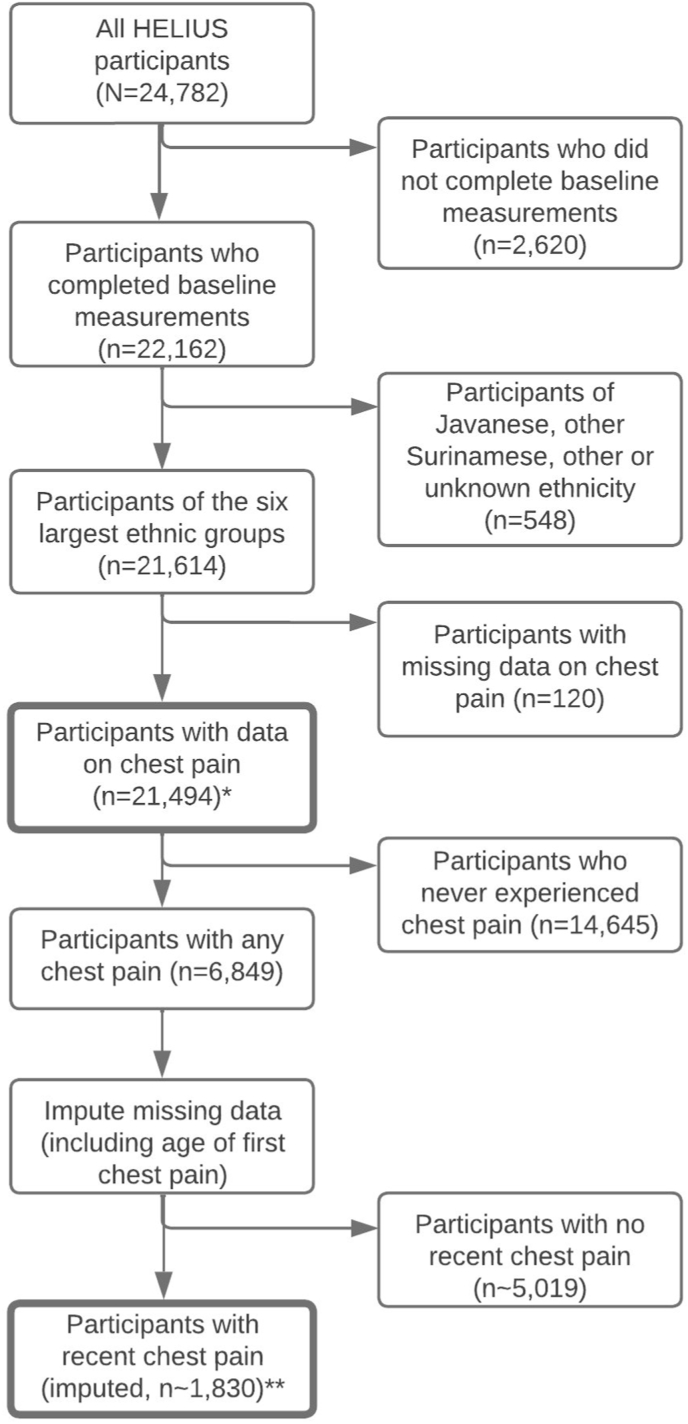
Flowchart of HELIUS participants included in this study. ∗HELIUS, HEalthy Life In an Urban Setting.

Of 24,782 participants, 22,162 completed the questionnaire and the physical examination. We excluded those of Javanese Surinamese (n = 233), other Surinamese (n = 267), other, and unknown ethnicity (n = 48) due to insufficient power for subgroup analyses. Next, we excluded participants with missing chest pain data (n = 120), leaving n = 21,494 participants (12,423 women and 9071 men) to study chest pain prevalence. Our subsequent analyses were restricted to those with recent chest pain (Fig. 1).

Due to the questionnaire routing, only people who answered positively on the question whether chest pain was elicited by physical exertion (40.6 % of participants with lifetime chest pain) were asked at what age they first experienced chest pain. Given the otherwise low power for further analyses (n = 810), we imputed missing data through multiple imputation via chained equations, using 5 imputations and 60 iterations. As we imputed the selection criterion ‘recent chest pain’, our sample size varied somewhat across imputed datasets, with a mean of 1830 participants.

### 2.6 Statistical analyses

Characteristics of the total population, and the prevalence and characteristics of chest pain are presented as medians [interquartile range (IQR)] or frequencies [%], for women and men in total, and by ethnicity. In age- (and ethnicity-)adjusted logistic regression analyses, we studied sex differences in chest pain in total, and across ethnic groups.

In those with recent chest pain, we described GP, cardiologist, and any specialist visits, in total and by ethnicity. First, in model 1, using logistic regression analyses, we studied sex differences in GP visits (adjusted for age only). This was done in the total population (age- and ethnicity-adjusted) and by ethnicity (age-adjusted only). Next, in model 2, we additionally adjusted for socioeconomic factors (educational level, employment status), associated symptoms (dyspnoea, fatigue), clinical parameters (awareness of hypertension, diabetes, hypercholesterolemia), and lifestyle factors (obesity, smoking), as sex- and ethnic differences in risk profiles may be related to HCSB.

Finally, in those with recent chest pain who visited their GP in the previous year (as in the Netherlands, patients are generally referred to specialists by GPs), we studied sex differences in cardiologist and any specialist visits (model 1 and 2). All analyses were performed in R version 3.14.0, with statistical significance determined at P < .05.

#### 2.6.1 Additional analyses

First, we studied sex differences in pulmonologists, internists and/or gastroenterologists, and mental health specialists separately. Next, we studied sex differences in supplementary diagnostics listed in general practice guidelines [19].

#### 2.6.2 Sensitivity analyses

To determine whether our definition of recent chest pain affected our results, we first limited our definition to chest pain reflective of typical AP, given that increased (perceived) severity may associate with HCSB. Second, we limited recent chest pain to a first episode of chest pain in the previous twelve months, as this may increase the likelihood that care contacts reported during the previous year were related to the experienced chest pain. Next, as prior CVD diagnoses may increase the likelihood that those with chest pain sought or received care, we studied sex differences in GP and cardiologist visits, excluding those with prior CVD. Finally, we additionally adjusted our models for GP and cardiologist visits for depressive symptoms, as studies suggest healthcare avoidance [23] and diagnostic overshadowing (the attribution of physical symptoms to psychological rather than physiological causes) [24] in those with mental health issues.

## 3 Results

Our sample consisted of 12,423 women and 9071 men. The median age [IQR] was 43.8 years [13.2] in women and 44.9 years [13.2] in men (Table 1). Similar proportions of women and men had a lower education, but more women than men had no paid employment. The prevalence of obesity, dyspnoea, fatigue, and awareness of hypertension appeared higher in women, while men had higher rates of smoking, and awareness of diabetes and hypercholesterolemia. Compared to the total population, those with recent chest pain were of similar ages, but had a lower SES, reported more associated symptoms, and had less favorable clinical parameters and lifestyle factors (Supplemental Table 2).

**Table 1:** Characteristics of women and men in the study, in total and by ethnicity.

	Total	Dutch	South Asian Surinamese	African Surinamese	Ghanaian	Turkish	Moroccan
**Women (n)**	12423	2474	1664	2523	1408	1969	2385
Median age [IQR]	45 [33, 54]	47 [32, 57]	48 [37, 56]	50 [40, 57]	45 [37, 51]	41 [30, 49]	39 [28, 50]
**Socioeconomic characteristics**							
Low education	5515 [44.8]	441 [17.9]	820 [49.6]	943 [37.6]	1008 [73.0]	1118 [57.4]	1185 [50.0]
Not employed	5573 [45.4]	681 [27.6]	683 [41.5]	939 [37.5]	645 [47.0]	1167 [60.3]	1458 [61.9]
**Associated symptoms**							
Dyspnoea	4029 [32.0]	565 [22.7]	663 [39.6]	827 [32.3]	214 [14.0]	869 [43.7]	880 [36.7]
Fatigue	4142 [33.6]	442 [17.9]	669 [40.4]	738 [29.5]	130 [9.4]	1077 [55.1]	1086 [45.7]
**Clinical parameters**							
Hypertension	3320 [27.0]	453 [18.3]	525 [31.8]	1020 [40.8]	501 [36.2]	422 [21.7]	399 [16.9]
Diabetes	1125 [9.1]	49 [2.0]	278 [16.8]	278 [11.1]	118 [8.5]	170 [8.7]	232 [9.8]
Hypercholesterolemia	1832 [15.0]	298 [12.2]	373 [23.4]	410 [16.7]	176 [12.7]	329 [16.9]	246 [10.5]
Depressive symptoms	2114 [17.2]	207 [8.4]	371 [22.5]	339 [13.6]	142 [10.3]	519 [26.6]	536 [22.7]
Prior CVD diagnosis	275 [2.3]	37 [1.5]	58 [3.6]	64 [2.6]	20 [1.5]	62 [3.3]	34 [1.5]
**Lifestyle factors**							
Obesity	3854 [31.1]	250 [10.1]	388 [23.3]	948 [37.6]	624 [44.4]	807 [41.0]	837 [35.1]
Smoking	2249 [18.2]	581 [23.6]	315 [19.0]	617 [24.6]	35 [2.5]	573 [29.3]	128 [5.4]
**Chest pain prevalence**							
Chest pain (lifetime)	4095 [33.0]^†††^	502 [20.3]	671 [40.3]	866 [34.3]^†††^	352 [25.0]^††^	776 [39.4]^†^	928 [38.9]^†††^
<2 year before baseline	563 [4.5]^††^	33 [1.3]^†^	95 [5.7]^†^	106 [4.2]	60 [4.3]^†^	131 [6.7]	138 [5.8]
<1 year before baseline	356 [2.9]	14 [0.6]	62 [3.7]	70 [2.8]	40 [2.8]	87 [4.4]	83 [3.5]
Median age first chest pain [IQR]	40 [25, 49]	45 [29, 54]	44 [30, 50]	40 [25, 50]	40 [30, 47]	36 [23, 45]	37 [23, 46]

**Men (n)**	9071	2086	1362	1610	892	1615	1506
Median age [IQR]	46 [34, 55]	48 [35, 59]	46 [33, 56]	51 [40, 58]	49 [41, 55]	42 [31, 50]	42 [32, 52]
**Socioeconomic characteristics**							
Low education	3849 [42.8]	352 [17.0]	613 [45.2]	756 [47.4]	540 [61.4]	882 [55.0]	706 [47.6]
Not employed	2804 [31.2]	513 [24.6]	461 [34.2]	567 [35.7]	257 [29.2]	498 [31.4]	508 [33.9]
**Associated symptoms**							
Dyspnoea	2092 [22.8]	393 [18.8]	435 [31.8]	298 [18.5]	83 [8.7]	481 [29.2]	402 [26.2]
Fatigue	1705 [18.9]	190 [9.1]	358 [26.3]	216 [13.4]	53 [6.0]	513 [31.9]	375 [25.1]
**Clinical parameters**							
Hypertension	2276 [25.3]	469 [22.5]	405 [29.8]	497 [31.1]	355 [40.2]	286 [18.0]	264 [17.7]
Diabetes	854 [9.5]	64 [3.1]	253 [18.6]	138 [8.6]	114 [12.9]	134 [8.4]	151 [10.1]
Hypercholesterolemia	1620 [18.5]	338 [16.6]	386 [30.4]	227 [14.6]	153 [17.7]	331 [20.9]	185 [12.5]
Depressive symptoms	1052 [11.7]	122 [5.9]	185 [13.6]	105 [6.6]	62 [7.1]	305 [19.2]	273 [18.3]
Prior CVD diagnosis	370 [4.2]	64 [3.1]	114 [8.7]	68 [4.3]	20 [2.4]	80 [5.1]	24 [1.7]
**Lifestyle factors**							
Obesity	1567 [17.3]	211 [10.1]	186 [13.7]	277 [17.2]	156 [17.5]	448 [27.8]	289 [19.2]
Smoking	2900 [32.1]	548 [26.3]	540 [39.8]	687 [42.9]	68 [7.7]	662 [41.3]	395 [26.4]
**Chest pain prevalence**^a^							
Lifetime chest pain	2634 [29.0]^†††^	471 [22.6]	508 [37.3]	385 [23.9]^†††^	183 [20.5]^††^	580 [35.9]^†^	507 [33.7]^†††^
<2 year before baseline	247 [2.7]^††^	19 [0.9]^†^	49 [3.6]^†^	37 [2.3]	13 [1.5]^†^	82 [5.1]	47 [3.1]
<1 year before baseline	157 [1.7]	12 [0.6]	30 [2.2]	22 [1.4]	7 [0.8]	54 [3.3]	32 [2.1]
Median age first chest pain [IQR]	40 [25, 49]	40 [24, 51]	40 [30, 50]	42 [26, 50]	45 [34, 50]	38 [25, 45]	38 [23, 48]

With the exception of age, all data are presented as n [%].

^†^p < .05, ^††^p < .01, ^†††^p < .001. IQR, Interquartile range; CVD, cardiovascular disease. This table is based on non-imputed data.

a Only sex differences in prevalence of lifetime and recent chest pain were tested, adjusted for age, and for age and ethnicity in the total population.

### 3.1 Chest pain prevalence

The lifetime prevalence of chest pain was 33 % in women and 29 % in men. Across all ethnic groups except native Dutch, women statistically significantly more often reported lifetime chest pain than men. The prevalence of chest pain ranged from 20.3 % in Dutch women to 40.3 % in South Asian Surinamese women, and 20.5 % in Ghanaian men to 37.3 % of South Asian Surinamese men. Chest pain characteristics varied somewhat by sex; e.g., 13.9 % of women versus 9.3 % of men had symptoms indicative of typical AP (Supplemental Table 3). The age- and ethnicity-adjusted prevalence of recent chest pain was significantly higher in women (4.5 %) than men (2.7 %), and patterns of sex differences were similar across most ethnic groups.

### 3.2 Differences in care contacts

**Fig. 2:**
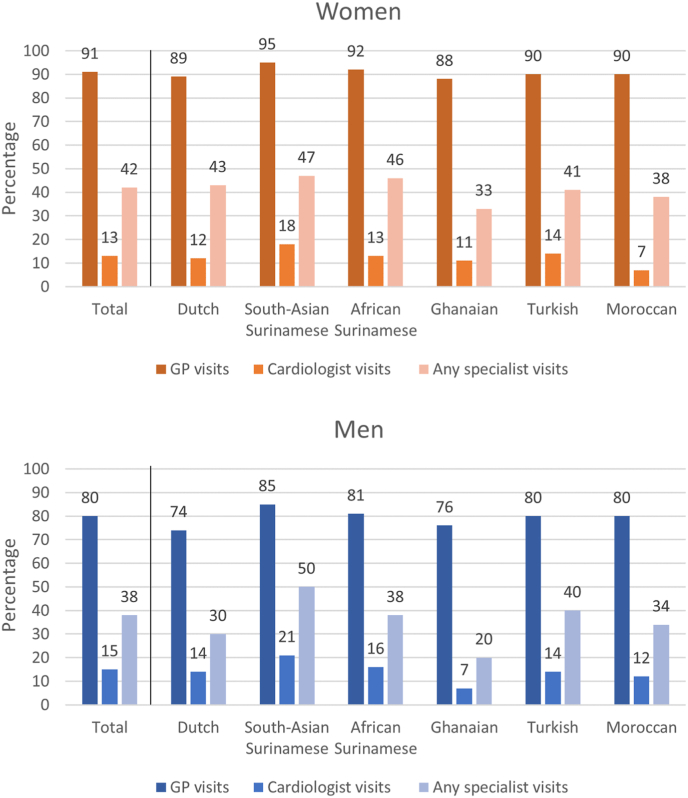
Proportion of care contacts for women and men, by ethnicity.

**Fig. 3:**
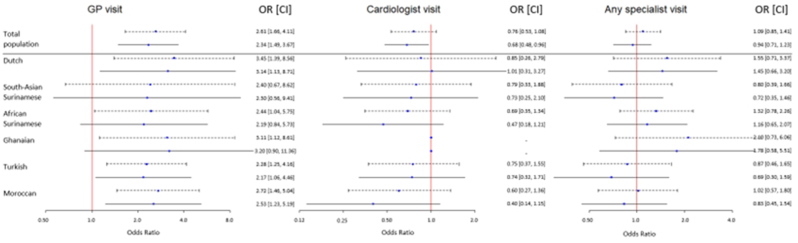
Odds ratios and confidence intervals of sex differences in GP, cardiologist, and any specialist visits. ∗GP, General Practitioner; OR, Odds Ratio; CI, Confidence Interval.

Among those with recent chest pain, more women (90.7 %) than men (79.8 %) reported recent GP visits (Fig. 2, Supplemental Table 4). This pattern was consistent across ethnic groups, with South Asian Surinamese women and men most often (94.6 % and 85.1 %) and Ghanaian women and Dutch men least often (88.0 % and 73.8 %) reporting having visited a GP. Women with recent chest pain had about two to three times higher adjusted odds of having visited a GP compared to men (Fig. 3).

In total, 12.5 % of women and 15.0 % of men reported a cardiologist visit, ranging from 7.2 % in Moroccan woman to 18.4 % in South Asian Surinamese women, and from 7.3 % in Ghanaian men to 21.1 % in South Asian Surinamese men (Fig. 2, Supplemental Table 4). Women were less likely to have visited a cardiologist than men, even when sex differences in risk profiles had been accounted for (Fig. 3). This was observed across most ethnic groups, albeit not significantly, with the exception of the Dutch and Ghanaian group. For the Ghanaians, however, the results should be interpreted cautiously due to the low power and broad confidence intervals. Despite fewer cardiologist visits, women and men were equally likely to have visited any specialist.

### 3.3 Additional analyses

Similar to cardiologist visits, women were less likely to have visited a pulmonologist than men (Supplemental Table 5). Contrarily, in particular in some ethnic groups, women appeared slightly more likely than men to have visited an internist and/or gastroenterologist, and a mental health specialist, yet these differences were not statistically significant. Moreover, women were more likely than men to have received supplementary diagnostics (Supplemental Table 6).

### 3.4 Sensitivity analyses

For the typical AP subgroup, sex differences in cardiologist visits underscored the estimated sex differences in those with any recent chest pain (Supplemental Table 7). The results in the subgroups with a first episode of chest pain in the previous twelve months, without prior CVD, as well as the analyses additionally adjusted for depressive symptoms, did not change our interpretation of the results for GP and cardiologist visits.

## 4 Discussion

Across most ethnic groups, women were more likely than men to report having experienced lifetime and recent chest pain. Among those with recent chest pain, women across all ethnic groups were more likely than men to have visited a GP. While women with recent chest pain in most groups were less likely to have visited a cardiologist, they were as likely as men to report having visited any specialist. This sex difference was also observed in the subgroup with AP. Sensitivity analyses, e.g., using different definitions of recent chest pain, did not affect our interpretation of these results.

We acknowledge several limitations to our study. First, due to our use of cross-sectional survey data, we cannot be certain whether the chest pain preceded care contacts, or if specialist visits resulted from a GP referral. Thus, we cannot draw any definite conclusions from the differences observed in our study.

A second limitation pertains to the operationalization of variables. First, while the Rose Angina Questionnaire has been validated for studying AP in general populations [25], the validity of the question on ‘any’ chest pain for identifying CVD (risk) in general populations may be limited, given the various nature and frequent occurrence of chest pain [[1], [2], [3], [4]]. Based on the questionnaire data, we could not distinguish between cardiac and non-cardiac, nor between acute and chronic chest pain. However, this information is essential to determine the severity of chest pain and the necessity and urgency of a GP or specialist visit. Thus, we cannot determine whether women and men in this study required GP or specialist care. Data on care contacts was also self-reported and may thus be subject to recall bias, as individuals may over- or under-report symptoms and whether they have visited a specialist, thereby affecting the reliability of our findings. In addition, the current data does not capture differences in the number of GP or cardiologist visits made, which may be important if women or men require more visits before care is initiated. For a more complete depiction of reasons for possible delays in care, and the potential impact on clinical outcomes for ethnically diverse women and men with chest pain, we recommend future research use linkage with general practice or hospital data to provide more insights into care pathways and diagnoses. Such work will allow exploration of mechanisms contributing to delays, including, e.g., frequency of visits, patients' description and communication of symtoms, the GP's interpretation of symptoms, or factors within the shared decision making process.

A final limitation pertains to our imputation strategy, specifically imputing missing data on the Rose Angina Questionnaire. The questionnaire aims to assess whether chest pain meets the criteria of e.g. typical AP, and thus, timing of chest pain was only asked in those who experienced chest pain elicited by exertion. This implies that missingness was not at random, meaning participants with missing data may differ from those who answered these questions, which could have affected our estimates in the imputed dataset. As proportions of recent chest pain were similar in our imputed (26.7 %) and non-imputed sample (30.6 %), we presume that imputation has not drastically altered our findings.

The prevalence of lifetime chest pain in our study was similar to prevalence rates reported in earlier studies in white European populations [1,10]. These studies found that women more often experienced chest pain than men [8,10]. While we did not find this pattern among native Dutch participants, this was pattern consistently observed across ethnic minority groups. This difference between the Dutch and other groups in our study may relate to the relatively lower prevalence of obesity in the Dutch in our study, which has been associated with cardiac [26] and non-cardiac [27] chest pain, and the mean age at which cardiovascular risk may reveal itself [13].

Our finding that about 10–20 % of women and men with recent chest pain had not visited their GP in the previous year indicates that not all individuals with symptoms suggestive of CVD seek care. While not all individuals with chest pain who did not visit a GP may require further diagnostics or treatment, as most care contacts for chest pain in those who do seek care do not concern a severe underlying condition [8,9], there is evidence to suggest some individuals with chest pain are missing out on necessary treatment. For instance, earlier work in the same cohort shows that 15–20 % of those with an unrecognized myocardial infarction reported having experienced chest pain, yet did not seek or receive care [28]. Missed or delayed diagnoses may have substantial negative consequences, including damaged heart tissue, worse cardiovascular outcomes, and an increased risk of mortality [29], as well as a broader societal economic impact when people in the working population are affected [30]. Given the clinical and societal consequences of missed and delayed diagnoses, differentiating between ‘harmless’ chest pain versus chest pain that requires (emergency) care remains an important area of interest with significant clinical implications [[31], [32], [33]].

In our diverse study population, including heterogeneity in language, culture, religion, migration history, and SES [18], there may be various reasons for not seeking care both in general, and for chest pain specifically. Based on qualitative work, barriers to care specific to chest pain may include factors such as poor symptom recognition and low perceived severity of symptoms [34], including differences in perceptions and interpretations of chest pain [35]. Barriers to care in general, not solely for potentially cardiac symptoms, may include, for instance, sex- and ethnic specific barriers [36]. Sex specific barriers to care included norms on femininity that describe women should prioritize caring for others over caring for themselves, and norms on masculinity that dictate that male toughness, and that seeking care could be perceived as weakness. Moreover, ethnic specific barriers to care may include language barriers and mistrust in healthcare services [37], and the use of religious-, spiritual-, or cultural healing practices versus seeking medical attention. Information on such barrier to care may be used for intervention development to promote HCSB in those at risk.

Moreover, our findings suggest sex differences in HCSB in those with chest pain, with men across all ethnic groups being two to three times less likely to have visited a GP than women. Our findings align with earlier work [38] that also found women with chest pain were more likely to have visited a GP than men [39]. These findings, however, contradict other work that reported longer patient delays in women [17]. This contradiction may be explained by our study's focus on chest pain as a typical cardiac symptom, whereas delays in care seeking in women are often attributed to ‘atypical’ symptom presentations [17]. Alternatively, our figures may mask differences, given that the higher proportions of GP visits in women may reflect other reasons for GP visits than chest pain, e.g., reproductive issues [38]. Nevertheless, even such visits may present an opportunity for discussion of other symptoms and initiation of care.

While women were more likely to have visited a GP, in most groups -except the Ghanaian and Dutch-they were less likely to have visited a cardiologist than men. We could not distinguish between cardiac and non-cardiac chest pain, nor do we know if people received a referral but did not visit a cardiologist for other reasons, e.g. due to financial concerns. Nevertheless, these findings align with other work that showed women were less frequently referred to cardiologists than men, while they equally often presented with cardiac chest pain [11]. For the Dutch population, we hypothesize that Dutch women may be better able to voice their concerns [40] and navigate the Dutch healthcare system, or may (subconsciously) be treated differently by their physician [41], compared to women of some ethnic minority groups.

Despite this sex difference in cardiologist visits, we found no sex differences in visits to any specialist. This related to a higher number of visits to other specialists in women, particularly gastroenterologists, internists, and mental health specialists. Similar patterns of sex differences in referrals have been reported in other work [42]. While we could not determine reasons for these differences in our study, we hypothesize it may partly reflect the GP's risk assessment, based on women's generally lower CVD risk compared to men [43] as well as on available diagnostic information. Women in our study more frequently reported having had supplementary diagnostics, and GPs will likely have taken the results of these diagnostics into account in their decision to (not) refer patients to a cardiologist. Nevertheless, our study suggests that the differences in referral patterns may not be completely justified based on a priori risk. In our data, the sex difference in cardiologist visits appeared even more pronounced among the subgroup with symptoms suggestive of typical AP. As typical AP has been associated with CVD [25], this would suggest some women at risk of CVD may indeed more frequently miss out on necessary care than men, implying a possible system delay.

Importantly, these sex differences in patterns of referrals were present across ethnic groups, including ethnic groups with a known high CVD burden. Women of Dutch origin with chest pain were equally likely as their male counterparts to have visited a cardiologist, yet, in particular women of some ethnic minority groups were less likely to be referred to a cardiologist, yet more likely than men to be referred to internist/gastro-enterologists or mental health specialists. These findings could suggest that chest pain may be interpreted differently in women and men of different ethnic groups.

Concluding, across most ethnic groups, we found a higher prevalence of chest pain in women than men. Moreover, our findings suggest sex differences in HCSB and referrals, as men were less likely to have visited a GP, while women were less likely to have visited a cardiologist. Combined, this suggests delays in care may occur at different points in the care trajectory for women and men. This suggests that different strategies may be implemented to improve adequate HCSB and referrals, including improving symptom recognition amongst the general population, as well as easily-accessible diagnostic testing to rule out or diagnose CVD in primary care. Future research is needed to determine the impact of these individuals not receiving timely care, explore modifiable risk factors for delays in care, and subsequently, develop interventions to improve the care process.

## CRediT authorship contribution statement

**Bryn Hummel:** Writing – original draft, Formal analysis, Conceptualization. **Ralf E. Harskamp:** Writing – review & editing, Conceptualization. **Annick Vester:** Writing – review & editing, Writing – original draft. **Henrike Galenkamp:** Writing – review & editing. **Paula M.C. Mommersteeg:** Writing – review & editing. **Irene G.M. van Valkengoed:** Writing – review & editing, Writing – original draft, Conceptualization.

## Data availability statement

The HELIUS data are owned by the Amsterdam University Medical Centers, location AMC in Amsterdam, The Netherlands. Any researcher can request the data by submitting a proposal to the HELIUS Executive Board as outlined at http://www.heliusstudy.nl/en/researchers/collaboration, by email: heliuscoordinator@amsterdamumc.nl. The HELIUS Executive Board will check proposals for compatibility with the general objectives, ethical approvals and informed consent forms of the HELIUS study. There are no other restrictions to obtaining the data and all data requests will be processed in the same manner.

## Conflict of interest

None declared.

## Figures and Tables

**figs1:**