# Supplementary material for: Chest pain in a multi-ethnic population: A community-based study on sex differences in chest pain prevalence and care contacts
Source: Int J Cardiol Cardiovasc Risk Prev. 2024 Dec 17;24:200361. doi: 10.1016/j.ijcrp.2024.200361 (PMC11728961; doi:10.1016/j.ijcrp.2024.200361)
Supplement: Multimedia component 1 [file mmc1.docx]

**Supplemental table 1. Definitions of variables in the study.**

| *Any chest pain* | Participants who answered positively to the question: “have you ever experienced chest pain or an uncomfortable feeling in your chest (excluding symptoms caused by a cold, asthma or heartburn)?”. |
| --- | --- |
| *Recent chest pain* | Participants who answered positively to the question: “have you ever experienced chest pain or an uncomfortable feeling in your chest (excluding symptoms caused by a cold, asthma or heartburn)?”, who reported a first episode of chest pain in the past two years before HELIUS data collection. This two-year period was chosen because care outcomes were reported in the questionnaire for the year before baseline. Recency was calculated by subtracting the age at which participants reported first experiencing chest pain from their age at baseline. We refer to as ‘recent chest pain’ for readability, while recognizing ‘recent’ may refer to a shorter time period in clinical practice. |
| *Typical AP* | Chest pain provoked by physical exertion, which disappeared within ten minutes after standing still and/or using sublingual nitrates. |
| *Recent GP visits* | Recent GP visits were defined as participants reporting having visited a GP, including phone consultations and home visits, but not phone consultations for repeat subscriptions or to book an appointment. With recent, we refer to visits in the previous twelve months before baseline, since this timeframe was available for all outcomes. |
| *Recent cardiologist visits* | Self-reported visits to a cardiologist in the previous twelve months before baseline, including outpatient treatment, yearly follow-up visits, phone consultations, and first aid, but not specialist care during hospital admissions or consultations to book an appointment |
| *Recent visits to other specialists* | As chest pain may also have a pulmonary, other physical, or psychological cause, we studied self-reported visits to other specialists that patients may be referred to for chest pain: pulmonologists, internists and/or gastroenterologists, and mental health specialists (psychotherapists, psychiatrists, psychologists, and social workers), in the previous twelve months before baseline. This included outpatient treatment, yearly follow-up visits, phone consultations, and first aid, but not specialist care during hospital admissions or consultations to book an appointment |
| *Supplementary diagnostics* | Participants reporting having had their blood pressure, blood glucose, or cholesterol measured by a healthcare professional in the previous twelve months. |
| *Age* | Age (in years) at the time of the data collection was determined from the date of birth derived from the municipality registry. |
| *Sex* | Sex (female or male) was derived from the municipality registry. We recognize that this does not capture the non-visible variation, e.g. intersex. Moreover, we use the terms ‘’women’’ and ‘’men’’ over the terms male and female to recognize that differences observed in our study may not only reflect biological factors (sex) but also sociocultural factors (gender). |
| *Ethnicity* | Ethnicity was defined based on participants’ and their parents’ country of birth. Participants were considered to belong to one of the included ethnic minority groups if they, and at least one parent, were born abroad, or if they were born in the Netherlands, but both their parents were born abroad. Surinamese participants were further classified according to self-reported ethnic origin into ‘African’, ‘South Asian’, ‘Javanese’, or ‘other’. Participants were considered to be of Dutch origin if they, and both their parents, were born in the Netherlands. |
| *Educational level* | Educational level was based on the highest obtained qualification in the Netherlands or the country of origin. This was classified into lower (including no or elementary education, lower vocational, and lower secondary education), and higher educational level (including intermediate or higher secondary schooling, intermediate or higher vocational schooling, and university). |
| *Employment status* | Employment status was categorised into employed and not currently employed, including not in the labor force, unemployed, and occupationally disabled. |
| *Dyspnea* | Dyspnea was measured by asking participants if they ever experienced shortness of breath, excluding dyspnea caused by a cold. |
| *Fatigue* | Fatigue was assessed by asking if participants experienced severe and/or long-term fatigue in the past year. |
| *Awareness of hypertension* | Awareness of hypertension was defined based on self-reported diagnosed hypertension, asked via the question: “has a medical professional ever diagnosed you with high blood pressure?”, and antihypertensive medication (Anateumical Therapeutic Codes (ATC) C02, C03, C07, C08, and C09). |
| *Awareness of diabetes* | Awareness of diabetes was defined based on self-reported diagnosed diabetes, asked via the question: “has a medical professional ever diagnosed you with high blood sugar?”, and glucose-lowering medication (ATC A10). |
| *Awareness of hypercholesterolemia* | Awareness of hypercholesterolemia was defined based on self-reported diagnosed hypercholesterolemia, asked via the question: “has a medical professional ever diagnosed you with high blood cholesterol?”, and cholesterol-lowering medication (C10). |
| Depressive symptoms | Depressive symptoms were measured by the Patient Health Questionnaire (PHQ-9), which measures how frequently, in the past two weeks, participants experienced depressive symptoms. PHQ-9 sum scores >9 were considered significant depressive symptoms. |
| Prior Cardiovascular disease (CVD) | Prior CVD diagnoses were defined as participants reporting a stroke or MI, or having had percutaneous coronary intervention or bypass surgery, more than two years before baseline measurements. This was computed by subtracting the age at which participants reported these events from their age at baseline. |
| *Obesity* | Obesity was defined as a Body Mass Index ≥30. Body Mass Index was calculated as weight (kg) divided by height squared (m^2^), with weight and height measured in duplicate, with the participant in light clothing, during physical examinations. |
| *Smoking status* | Smoking status was based on self-report, distinguishing between current smokers and non-smokers (including past and never smokers). |

**Supplemental table 2. Characteristics of women and men with recent chest pain, in total and by ethnicity.**

|  | Total | Dutch | South Asian Surinamese | African Surinamese | Ghanaian | Turkish | Moroccan |
| --- | --- | --- | --- | --- | --- | --- | --- |
| **Women (n)** | 1167 | 141 | 190 | 229 | 110 | 228 | 269 |
| Median age [IQR] | 44 [32, 52] | 51 [39, 57] | 51 [41, 58] | 50 [38, 55] | 46 [36, 51] | 40 [27, 47] | 41 [29, 50] |
|  |  |  |  |  |  |  |  |
| **Sociodemographic characteristics** |  |  |  |  |  |  |  |
| Low education | 581 [49.8] | 40 [28.4] | 101 [52.9] | 92 [40.1] | 82 [74.4] | 129 [56.4] | 139 [51.5] |
| Not employed | 605 [51.9] | 49 [34.6] | 87 [45.8] | 92 [40.2] | 57 [51.7] | 145 [63.5] | 176 [65.4] |
|  |  |  |  |  |  |  |  |
| **Associated symptoms** |  |  |  |  |  |  |  |
| Dyspnoea | 612 [52.5] | 63 [44.5] | 111 [58.2] | 112 [48.9] | 33 [29.9] | 151 [64.6] | 146 [54.4] |
| Fatigue | 610 [52.3] | 46 [32.4] | 111 [58.2] | 100 [43.4] | 23 [20.9] | 164 [72.1] | 167 [62.1] |
|  |  |  |  |  |  |  |  |
| **Clinical parameters** |  |  |  |  |  |  |  |
| Hypertension | 362 [31.0] | 34 [24.3] | 74 [38.7] | 101 [44.2] | 44 [39.8] | 59 [26.0] | 50 [18.7] |
| Diabetes | 122 [10.5] | 4 [2.5] | 36 [18.7] | 26 [11.4] | 11 [9.6] | 20 [8.7] | 27 [10.0] |
| Hypercholesterolemia | 228 [19.6] | 27 [19.0] | 61 [32.4] | 45 [19.6] | 17 [15.8] | 43 [18.9] | 35 [13.0] |
| Depressive symptoms | 313 [26.9] | 21 [14.6] | 63 [33.1] | 49 [21.2] | 16 [14.1] | 81 [35.6] | 85 [31.4] |
| Prior CVD diagnosis | 37 [3.2] | 3 [1.8] | 8 [4.3] | 6 [2.6] | 5 [4.1] | 11 [4.6] | 5 [2.0] |
|  |  |  |  |  |  |  |  |
| **Lifestyle factors** |  |  |  |  |  |  |  |
| Obesity | 386 [33.1] | 17 [12.3] | 42 [22.3] | 89 [39.0] | 43 [39.2] | 90 [39.6] | 104 [38.5] |
| Smoking | 245 [21.0] | 41 [29.1] | 34 [17.7] | 67 [29.3] | 2 [1.7] | 81 [35.4] | 21 [7.9] |
|  |  |  |  |  |  |  |  |
| **Men (n)** | 663 | 115 | 123 | 105 | 41 | 157 | 123 |
| Median age [IQR] | 45 [31, 53] | 40 [25, 58] | 45 [38, 55] | 47 [39, 58] | 50 [41, 52] | 41 [27, 50] | 49 [34, 54] |
|  |  |  |  |  |  |  |  |
| **Sociodemographic characteristics** |  |  |  |  |  |  |  |
| Low education | 302 [45.6] | 20 [17.6] | 58 [47.3] | 53 [50.9] | 24 [59.6] | 88 [55.9] | 57 [47.6] |
| Not employed | 235 [35.4] | 30 [26.5] | 48 [39.3] | 39 [37.6] | 14 [34.7] | 53 [34.1] | 49 [39.9] |
|  |  |  |  |  |  |  |  |
| **Associated symptoms** |  |  |  |  |  |  |  |
| Dyspnoea | 276 [41.5] | 39 [33.6] | 60 [49.3] | 34 [32.5] | 8 [30.4] | 76 [48.2] | 58 [27.4] |
| Fatigue | 232 [35.0] | 16 [14.3] | 51 [41.6] | 26 [25.0] | 3 [7.8] | 84 [53.4] | 52 [41.8] |
|  |  |  |  |  |  |  |  |
| **Clinical parameters** |  |  |  |  |  |  |  |
| Hypertension | 189 [28.5] | 29 [25.3] | 42 [33.9] | 35 [33.0] | 20 [48.6] | 33 [21.3] | 31 [24.9] |
| Diabetes | 74 [11.2] | 3 [2.3] | 26 [20.9] | 7 [6.4] | 7 [16.8] | 16 [9.9] | 17 [13.7] |
| Hypercholesterolemia | 157 [23.7] | 24 [21.3] | 41 [33.1] | 17 [18.2] | 8 [18.4] | 42 [26.8] | 24 [19.2] |
| Depressive symptoms | 138 [20.8] | 11 [9.6] | 25 [20.4] | 14 [13.1] | 4 [8.6] | 45 [28.6] | 40 [32.4] |
| Prior CVD diagnosis | 30 [4.5] | 6 [5.2] | 9 [7.2] | 4 [4.2] | 1 [2.4] | 4 [2.5] | 6 [4.6] |
|  |  |  |  |  |  |  |  |
| **Lifestyle factors** |  |  |  |  |  |  |  |
| Obesity | 122 [18.4] | 12 [10.8] | 18 [14.7] | 17 [15.8] | 5 [11.0] | 40 [25.5] | 31 [24.7] |
| Smoking | 254 [38.3] | 32 [27.8] | 49 [39.7] | 54 [51.4] | 3 [6.1] | 78 [49.8] | 39 [31.4] |

With the exception of age, all data are presented as n [%], averaged across imputed datasets. Age is presented as the median [IQR] age across imputed datasets. IQR, interquartile range; CVD, cardiovascular disease. This table is based on imputed data.

**Supplemental table 3. Chest pain characteristics in women and men who reported lifetime chest pain, in total and by ethnicity.**

|  | Total | Dutch | South Asian Surinamese | African Surinamese | Ghanaian | Turkish | Moroccan |
| --- | --- | --- | --- | --- | --- | --- | --- |
| **Women (n)** | 4095 | 502 | 671 | 866 | 352 | 776 | 928 |
| Typical AP^a^ | 578 [13.9] | 34 [6.8] | 106 [15.8] | 12 [1.4] | 47 [13.4] | 133 [17.1] | 131 [14.1] |
| Pain elicited by exertion^a^ | 1813 [43.6] | 95 [18.9] | 303 [45.2] | 369 [42.6] | 163 [46.3] | 426 [54.9] | 457 [49.2] |
| Pain elicited by walking^b^ | 849 [46.8] | 57 [60.0] | 148 [48.8] | 190 [51.5] | 76 [46.6] | 165 [38.7] | 213 [46.6] |
|  |  |  |  |  |  |  |  |
| **When this happens, do you^b^** |  |  |  |  |  |  |  |
| Continue walking/cycling | 442 [24.4] | 39 [41.1] | 80 [26.4] | 94 [25.5] | 26 [16.0] | 85 [20.0] | 118 [25.8] |
| Slow down | 1248 [68.8] | 46 [48.4] | 198 [65.3] | 253 [68.6] | 122 [74.8] | 315 [73.9] | 314 [68.7] |
| Use sublingual nitrates | 18 [1.0] | 2 [2.1] | 4 [1.3] | 2 [0.5] | 3 [1.8] | 3 [0.7] | 4 [0.9] |
| Slow down and use sublingual nitrates | 59 [3.3] | 4 [4.2] | 13 [4.3] | 11 [3.0] | 9 [5.5] | 14 [3.3] | 8 [1.8] |
|  |  |  |  |  |  |  |  |
| **What happens when you do this?^c^** |  |  |  |  |  |  |  |
| Pain continues | 402 [30.3] | 5 [9.6] | 59 [27.4] | 67 [25.2] | 44 [32.8] | 126 [38.0] | 101 [31.0] |
| Pain stops within 10 minutes | 578 [43.6] | 34 [65.4] | 106 [49.3] | 127 [47.7] | 47 [35.1] | 133 [40.1] | 131 [40.2] |
| Pain stops after 10 minutes | 295 [22.3] | 7 [13.5] | 43 [20.0] | 65 [24.4] | 37 [27.6] | 65 [19.6] | 78 [23.9] |
|  |  |  |  |  |  |  |  |
| **Location of chest pain^b^** |  |  |  |  |  |  |  |
| Upper sternum | 873 [48.2] | 49 [51.6] | 139 [45.9] | 186 [45.5] | 98 [60.1] | 193 [45.3] | 208 [45.5] |
| Lower sternum | 391 [21.6] | 17 [17.9] | 75 [24.8] | 86 [23.3] | 42 [25.8] | 98 [23.0] | 73 [16.0] |
| Left anterior chest | 654 [36.1] | 20 [21.1] | 137 [45.2] | 127 [34.4] | 30 [18.4] | 178 [41.8] | 162 [35.4] |
| Left arm | 271 [14.9] | 10 [10.1] | 48 [15.8] | 56 [15.2] | 15 [9.2] | 85 [20.0] | 57 [12.5] |
| Other | 224 [12.4] | 11 [11.6] | 45 [14.9] | 49 [13.3] | 14 [8.6] | 49 [11.5] | 56 [12.3] |
|  |  |  |  |  |  |  |  |
| **Men (n)** | 2634 | 471 | 508 | 385 | 183 | 580 | 507 |
| Typical AP* | 246 [9.3] | 33 [7.0] | 57 [11.2] | 35 [9.1] | 13 [7.1] | 65 [11.2] | 43 [8.5] |
| Pain elicited by exertion ^a^ | 965 [36.6] | 86 [18.3] | 209 [41.1] | 125 [32.5] | 65 [35.5] | 287 [49.5] | 193 [38.1] |
| Pain elicited by walking^b^ | 502 [52.0] | 54 [62.8] | 113 [54.1] | 67 [53.6] | 40 [61.5] | 137 [47.7] | 91 [47.2] |
|  |  |  |  |  |  |  |  |
| **When this happens, do you: ^b^** |  |  |  |  |  |  |  |
| Continue walking/cycling | 327 [33.9] | 35 [40.7] | 64 [30.6] | 49 [39.2] | 25 [38.5] | 94 [32.8] | 60 [31.1] |
| Slow down | 552 [57.2] | 44 [51.2] | 116 [55.5] | 65 [52.0] | 37 [56.9] | 172 [59.9] | 118 [61.1] |
| Use sublingual nitrates | 20 [2.1] | 1 [1.2] | 8 [3.8] | 3 [2.4] | 1 [1.5] | 3 [1.0] | 4 [2.1] |
| Slow down and use sublingual nitrates | 51 [5.3] | 6 [7.0] | 19 [9.1] | 6 [4.8] | 1 [1.5] | 12 [4.2] | 7 [3.6] |
|  |  |  |  |  |  |  |  |
| **What happens when you do this?^c^** |  |  |  |  |  |  |  |
| Pain continues | 195 [31.1] | 11 [21.6] | 44 [30.8] | 19 [25.7] | 17 [43.6] | 67 [35.8] | 37 [28.7] |
| Pain stops within 10 minutes | 246 [39.5] | 33 [64.7] | 57 [39.9] | 35 [47.3] | 13 [33.3] | 65 [34.8] | 43 [33.3] |
| Pain stops after 10 minutes | 158 [25.4] | 6 [11.8] | 39 [27.3] | 16 [21.6] | 7 [17.9] | 45 [24.1] | 45 [34.9] |
|  |  |  |  |  |  |  |  |
| **Location of chest pain^b^** |  |  |  |  |  |  |  |
| Upper sternum | 408 [42.3] | 35 [40.7] | 85 [40.7] | 62 [49.6] | 31 [47.7] | 119 [41.5] | 76 [39.4] |
| Lower sternum | 208 [21.6] | 17 [19.8] | 38 [18.2] | 23 [18.4] | 15 [23.1] | 74 [25.8] | 41 [21.2] |
| Left anterior chest | 408 [42.3] | 41 [47.7] | 105 [50.2] | 45 [36.0] | 22 [33.8] | 113 [39.4] | 82 [42.5] |
| Left arm | 146 [15.1] | 12 [14.0] | 26 [12.4] | 12 [9.6] | 7 [10.8] | 56 [19.5] | 33 [17.1] |
| Other | 124 [12.8] | 7 [8.1] | 20 [9.6] | 22 [17.6] | 12 [18.5] | 33 [11.5] | 30 [15.5] |

^a^ Percentages of those who experienced lifetime chest pain. ^b^ Percentages of those who experienced lifetime chest pain elicited by exertion. ^c^ Percentages of those who experienced lifetime chest pain elicited by exertion, who then slowed down and/or took sublingual nitrates. AP, Angina Pectoris. This table is based on non-imputed data.

**Supplemental table 4. Recent care contacts, in women and men with recent chest pain, in total and by ethnicity.**

|  | Total | Dutch | South Asian Surinamese | African Surinamese | Ghanaian | Turkish | Moroccan |
| --- | --- | --- | --- | --- | --- | --- | --- |
| **Women** | 1167 | 141 | 190 | 229 | 110 | 228 | 269 |
| *GP visit* | 1059 [90.7] | 125 [88.5] | 180 [94.6] | 210 [91.8] | 97 [88.0] | 204 [89.6] | 243 [90.3] |
| *Any specialist visit* | 485 [41.7] | 60 [42.6] | 91 [46.8] | 103 [45.6] | 36 [33.4] | 91 [40.6] | 104 [38.4] |
| *Cardiologist* | 146 [12.5] | 17 [12.0] | 35 [18.4] | 30 [13.2] | 12 [10.8] | 32 [14.0] | 20 [7.2] |
| Pulmonologist | 57 [4.8] | 6 [4.0] | 14 [7.5] | 9 [4.1] | 6 [5.0] | 13 [5.7] | 9 [3.2] |
| Internist/gastroenterologist | 164 [14.1] | 15 [10.6] | 33 [17.3] | 36 [15.8] | 9 [8.0] | 43 [18.8] | 29 [10.6] |
| Mental health specialist | 281 [24.1] | 37 [26.3] | 46 [24.4] | 58 [25.2] | 22 [19.5] | 47 [20.7] | 71 [26.5] |
| Care received abroad | 141 [12.1] | 0 [0.0] | 9 [4.7] | 16 [7.1] | 12 [10.6] | 71 [31.0] | 33 [12.3] |
|  |  |  |  |  |  |  |  |
| Any supplementary diagnostic test | 798 [68.4] | 84 [60.0] | 150 [79.1] | 171 [74.7] | 82 [74.7] | 147 [64.4] | 163 [60.7] |
| BP measurement | 754 [64.6] | 81 [57.7] | 143 [75.1] | 164 [71.4] | 82 [74.1] | 134 [58.7] | 151 [56.2] |
| BG measurement | 429 [36.7] | 39 [27.6] | 99 [51.8] | 96 [41.7] | 38 [34.8] | 77 [33.7] | 81 [30.0] |
| TC measurement | 309 [26.5] | 32 [22.8] | 67 [35.3] | 75 [32.8] | 37 [33.9] | 57 [24.8] | 41 [15.2] |
|  |  |  |  |  |  |  |  |
| **Men** | 663 | 115 | 123 | 105 | 41 | 157 | 123 |
| *GP visit* | 529 [79.8] | 86 [73.8] | 105 [85.1] | 85 [80.7] | 31 [75.5] | 126 [80.4] | 98 [79.7] |
| *Any specialist visit* | 250 [37.6] | 34 [30.0] | 63 [50.1] | 41 [37.9] | 9 [20.2] | 62 [40.4] | 43 [34.4] |
| *Cardiologist* | 99 [15.0] | 17 [14.4] | 26 [21.1] | 17 [15.8] | 3 [7.3] | 22 [14.3] | 15 [12.2] |
| Pulmonologist | 41 [6.2] | 5 [4.4] | 11 [9.2] | 5 [4.5] | 1 [2.0] | 12 [7.7] | 8 [6.1] |
| Internist/gastroenterologist | 70 [10.5] | 11 [9.9] | 17 [13.5] | 9 [8.3] | 2 [4.9] | 20 [12.5] | 12 [9.3] |
| Mental health specialist | 126 [19.1] | 17 [14.4] | 25 [20.4] | 21 [19.9] | 5 [11.0] | 32 [20.7] | 27 [21.9] |
| Care received abroad | 79 [11.9] | 0 [0.0] | 9 [6.9] | 7 [6.4] | 5 [11.8] | 46 [29.6] | 13 [10.3] |
|  |  |  |  |  |  |  |  |
| Any supplementary diagnostic test | 389 [58.6] | 55 [48.0] | 80 [65.0] | 75 [71.9] | 29 [69.8] | 78 [49.8] | 72 [58.2] |
| BP measurement | 372 [56.1] | 52 [45.4] | 78 [63.0] | 74 [70.5] | 28 [67.8] | 75 [47.8] | 66 [53.6] |
| BG measurement | 200 [30.1] | 21 [18.2] | 55 [44.2] | 35 [33.7] | 14 [34.7] | 37 [23.7] | 38 [30.8] |
| TC measurement | 186 [28.1] | 22 [19.2] | 50 [40.8] | 32 [30.3] | 14 [34.3] | 38 [24.4] | 30 [24.3] |

Prevalence is presented as the average n [%], across imputed datasets. GP, general practitioner; BP, blood pressure; BG, blood glucose; TC, total cholesterol. This table is based on imputed data.

**Supplemental table 5. Additional analyses of sex differences in pulmonologist, internist/gastroenterologist, and mental health specialist visits.**

|  | Pulmonologist | | | Internist and/or gastroenterologist | | | | | Mental health specialist | | | | | |
| --- | --- | --- | --- | --- | --- | --- | --- | --- | --- | --- | --- | --- | --- | --- |
|  | OR | 95%CI | p-value | OR | 95%CI | p-value | | OR | | 95%CI | | p-value | |  |
| Overall population |  |  |  |  |  | |  | |  | |  | |  | |
| Model 1 | 0.67 | [0.38, 1.17] | .154 | 1.27 | [0.87, 1.86] | | .213 | | 1.26 | | [0.83, 1.89] | | .250 | |
| Model 2 | 0.49 | [0.27, 0.90] | .023 | 1.10 | [0.71, 1.71] | | .645 | | 1.06 | | [0.70, 1.63] | | .759 | |
|  |  |  |  |  |  | |  | |  | |  | |  | |
| Dutch |  |  |  |  |  | |  | |  | |  | |  | |
| Model 1 | 0.78 | [0.19, 3.17] | .722 | 1.08 | [0.33, 3.53] | | .892 | | 2.49 | | [0.95, 6.48] | | .061 | |
| Model 2 | 0.54 | [0.09, 3.09] | .479 | 0.82 | [0.23, 2.87] | | .742 | | 2.23 | | [0.80, 6.27] | | .120 | |
|  |  |  |  |  |  | |  | |  | |  | |  | |
| South Asian Surinamese |  |  |  |  |  | |  | |  | |  | |  | |
| Model 1 | 0.65 | [0.19, 2.30] | .485 | 1.02 | [0.51, 2.07] | | .951 | | 1.18 | | [0.47, 2.98] | | .703 | |
| Model 2 | 0.41 | [0.12, 1.33] | .134 | 0.95 | [0.43, 2.06] | | .891 | | 1.17 | | [0.46, 2.97] | | .724 | |
|  |  |  |  |  |  | |  | |  | |  | |  | |
| African Surinamese |  |  |  |  |  | |  | |  | |  | |  | |
| Model 1 | 0.69 | [0.16, 2.91] | .595 | 1.91 | [0.57, 6.43] | | .272 | | 1.39 | | [0.67, 2.87] | | .362 | |
| Model 2 | 0.54 | [0.08, 3.92] | .517 | 1.63 | [0.44, 6.01] | | .436 | | 1.38 | | [0.60, 3.17] | | .432 | |
|  |  |  |  |  |  | |  | |  | |  | |  | |
| Ghanaian |  |  |  |  |  | |  | |  | |  | |  | |
| Model 1 | - | [0.00, INF] | .995 | 1.70 | [0.19, 15.62] | | .626 | | 2.01 | | [0.50, 8.11] | | .314 | |
| Model 2 | - | [0.00, INF] | .998 | 1.42 | [0.07, 28.74] | | .810 | | 1.77 | | [0.26, 12.10] | | .538 | |
|  |  |  |  |  |  | |  | |  | |  | |  | |
| Turkish |  |  |  |  |  | |  | |  | |  | |  | |
| Model 1 | 0.65 | [0.22, 1.90] | .425 | 1.61 | [0.80, 3.22] | | .179 | | 0.82 | | [0.39, 1.72] | | .587 | |
| Model 2 | 0.44 | [0.15, 1.28] | .132 | 1.47 | [0.61, 3.51] | | .374 | | 0.58 | | [0.25, 1.31] | | .177 | |
|  |  |  |  |  |  | |  | |  | |  | |  | |
| Moroccan |  |  |  |  |  | |  | |  | |  | |  | |
| Model 1 | 0.43 | [0.10, 1.86] | .245 | 0.87 | [0.31, 2.45] | | .781 | | 1.13 | | [0.64, 1.99] | | .663 | |
| Model 2 | 0.49 | [0.06, 3.73] | .466 | 0.81 | [0.25, 2.56] | | .702 | | 1.08 | | [0.56, 2.05] | | .823 | |

Model 1 is adjusted for age and ethnicity. Model 2 is additionally adjusted for associated symptoms, socioeconomic factors, clinical parameters, and lifestyle factors. CVD, Cardiovascular disease; OR, Odds Ratio (women versus men); CI, Confidence Interval.

**Supplemental table 6. Additional analyses of sex differences in supplementary diagnostics.**

|  | Supplementary diagnostics | | |
| --- | --- | --- | --- |
|  | OR | 95%CI | p-value |
| Overall population |  |  |  |
| Model 1 | 1.51 | [1.10, 2.07] | .012 |
| Model 2 | 1.48 | [1.11, 1.96] | .007 |
|  |  |  |  |
| Dutch |  |  |  |
| Model 1 | 1.80 | [0.88, 3.68] | .103 |
| Model 2 | 1.83 | [0.86, 3.90] | .113 |
|  |  |  |  |
| South Asian Surinamese |  |  |  |
| Model 1 | 2.00 | [1.02, 3.91] | .044 |
| Model 2 | 1.97 | [0.83, 4.64] | .118 |
|  |  |  |  |
| African Surinamese |  |  |  |
| Model 1 | 1.03 | [0.39, 2.74] | .949 |
| Model 2 | 0.79 | [0.29, 2.21] | .645 |
|  |  |  |  |
| Ghanaian |  |  |  |
| Model 1 | 1.26 | [0.24, 6.56] | .770 |
| Model 2 | 0.75 | [0.10, 5.73] | .766 |
|  |  |  |  |
| Turkish |  |  |  |
| Model 1 | 1.66 | [0.97, 2.82] | .062 |
| Model 2 | 1.98 | [1.06, 3.68] | .032 |
|  |  |  |  |
| Moroccan |  |  |  |
| Model 1 | 1.35 | [0.78, 2.33] | .284 |
| Model 2 | 1.31 | [0.64, 2.66] | .445 |

Model 1 is adjusted for age and ethnicity. Model 2 is additionally adjusted for associated symptoms, socioeconomic factors, clinical parameters, and lifestyle factors. OR, Odds Ratio (women versus men); CI, Confidence Interval.

**Supplemental table 7. Sensitivity analyses of sex differences in GP and cardiologist visits.**

|  | GP visit | | | Cardiologist visit | | |
| --- | --- | --- | --- | --- | --- | --- |
|  | OR | 95%CI | p-value | OR | 95%CI | p-value |
| Subgroup with a first chest pain in the previous twelve months |  |  |  |  |  |  |
| Model 1 | 2.65 | [1.80, 3.89] | <.001 | - | - | - |
| Model 2 | 2.43 | [1.56, 3.76] | <.001 | - | - | - |
|  |  |  |  |  |  |  |
| Subgroup with chest pain that fits the criteria of typical AP |  |  |  |  |  |  |
| Model 1 | 3.01 | [1.36, 6.66] | .007 | 0.30 | [0.13, 0.70] | .005 |
| Model 2 | 2.94 | [1.13, 7.67] | .028 | 0.22 | [0.08, 0.58] | .002 |
|  |  |  |  |  |  |  |
| Subgroup without prior CVD |  |  |  |  |  |  |
| Model 1 | 2.59 | [1.59, 4.22] | .001 | 0.82 | [0.56, 1.21] | .301 |
| Model 2 | 2.30 | [1.41, 3.75] | .003 | 0.73 | [0.51, 1.06] | .097 |
|  |  |  |  |  |  |  |
| Total population, additionally adjusted for depressive symptoms |  |  |  |  |  |  |
| Model 2 | 2.34 | [1.49, 3.68] | .001 | 0.68 | [0.48, 0.96] | .030 |

Model 1 is adjusted for age and ethnicity. Model 2 is additionally adjusted for associated symptoms, socioeconomic factors, clinical parameters, and lifestyle factors. GP, general practitioner, AP, angina pectoris; CVD, Cardiovascular disease; OR, Odds Ratio; CI, Confidence Interval.

**Supplemental Figure 1. Timeline recent chest pain and care outcomes.**

***CVD, Cardiovascular disease.**
